# Major Outbreak of Hepatitis A Associated with Orange Juice among Tourists, Egypt, 2004

**DOI:** 10.3201/eid1301.060487

**Published:** 2007-01

**Authors:** Christina Frank, Jan Walter, Marion Muehlen, Andreas Jansen, Ulrich van Treeck, Anja M. Hauri, Iris Zoellner, Magda Rakha, Marina Hoehne, Osamah Hamouda, Eckart Schreier, Klaus Stark

**Affiliations:** * Robert Koch Institute, Berlin, Germany; † Institute of Public Health, State of North-Rhine Westphalia, Münster, Germany; ‡ Hesse State Health Office, State of Hesse, Dillenburg, Germany; § State Health Authority, State of Baden-Wuerttemberg, Stuttgart, Germany;; ¶ Ministry of Health and Population, Cairo, Egypt

**Keywords:** immunization, travel medicine, hepatitis A, Egypt, disease outbreaks, food microbiology, dispatch

## Abstract

In 2004, a major outbreak of hepatitis A among tourists returning from Egypt involved 351 case-patients from 9 European countries who were infected with a single strain (genotype 1b). The case-control study identified orange juice as the most likely infection vehicle. Vaccination against hepatitis A virus is strongly recommended before travel to disease-endemic areas.

Nonimmune travelers to hepatitis A (HA)–endemic countries carry a substantial risk of acquiring the disease, yet little is known about the epidemiology of HA virus (HAV) infection in travel-associated outbreaks. In Germany, approximately half of the 1,400–2,300 cases of laboratory-confirmed HA reported annually since 2001 were acquired abroad. In mid-August 2004, infectious disease surveillance in Germany showed a strong increase of HAV infections in tourists returning from Egypt, where HA is highly endemic (*1**,**2*). The overwhelming majority had stayed at hotel X in the Red Sea resort of Hurghada. Prevention measures were implemented at the hotel (e.g., HA vaccination of guests). An outbreak investigation was carried out.

## The Study

The line listing of HA patients included persons with laboratory evidence of recent HAV infection (anti-HAV immunoglobulin M [IgM]) who stayed at hotel X after June 1, 2004. Also listed were hotel guests with HA disease (jaundice, elevated liver enzyme levels), without laboratory confirmation, who had traveled with persons with laboratory-confirmed cases.

A case-control study was performed among hotel X guests >17 years of age residing in 3 German states. The time span between the earliest arriving case-patient's last day at hotel X and the latest arriving case-patient's first day there was defined as the "minimum period of transmission" (MPT). Case-patients came from the line listing. Healthy hotel guests who stayed at the hotel during the MPT who had neither been vaccinated against HAV nor previously infected with HAV were eligible as controls. Telephone interviews were conducted with a standardized questionnaire that elicited information on demographic factors, foods and drink consumption, and participation in recreational activities such as swimming, day trips, etc.

For statistical analysis, exposures were dichotomized into "ever" versus "never," and the "number of days exposed" was calculated. Univariate analysis (χ^2^ tests) and multiple logistic regression were performed (SPSS version 12.0.2, SPSS Inc., Chicago, IL, USA); p<0.05 was considered statistically significant. The Egyptian authorities' investigation included testing all hotel employees for HAV antibodies (IgM, IgG), and scrutiny of food suppliers. Serum samples were obtained from German case-patients for testing by reverse transcription–PCR (RT-PCR) in the VP1–2A junction and sequencing of a 160-bp-long PCR fragment of the VP1 region.

**Figure 1:**
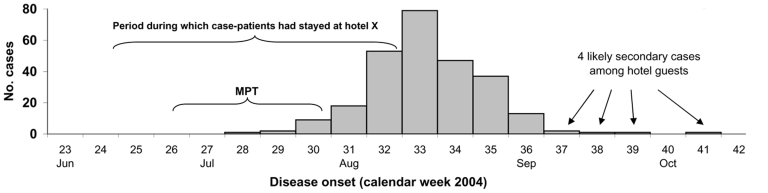
Epidemic curve: distribution of dates of disease onset for outbreak-associated hepatitis A case-patients from Germany (n = 264), and minimum period during which hepatitis A virus transmission occurred (MTP).

The outbreak lasted from July 10 to September 8, 2004 (Figure 1). A total of 351 case-patients made up this outbreak: 271 primary and 7 secondary infections in Germany, plus 60 primary infections reported to the national public health institutes in 8 other European countries. Austria recorded a secondary outbreak with 13 cases caused by an infected food handler who had stayed at hotel X (*3*). In preceding years, in Germany only 2–8 HA cases were reported after travel to Egypt with disease onset between July 10 and September 8.

Of the German reported primary cases, overt clinical HA developed in 263 (97%) persons. Case-patients were 2 to 67 years of age (median 34 years) and 54% were male. Overall, 47% of case-patients were hospitalized. Risk of hospitalization rose with increasing age (p for trend = 0.001). The MPT lasted from June 24 to July 23 (Figure 1). No more than 52% of the case-patients had stayed together at the hotel on any single day. Case-patients had stayed at hotel X from 6 to 21 days, and 70% had stayed 13 days or longer.

Sixty-nine HA case-patients (60% response among the 115 case-patients in the 3 states) and 36 controls were included in the statistical analysis. Eighty-seven percent of the case-patients reported absence from work for 3 to 56 days (median 26 days), and 54% of the case-patients were hospitalized for 2 to 25 days (median 9 days).

**Figure 2:**
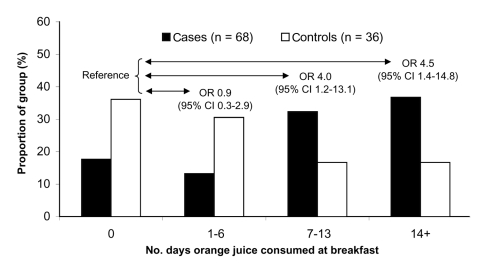
Days of orange juice consumption among hepatitis A patients and controls. OR, odds ratio; CI, confidence interval.

**Figure 3:**
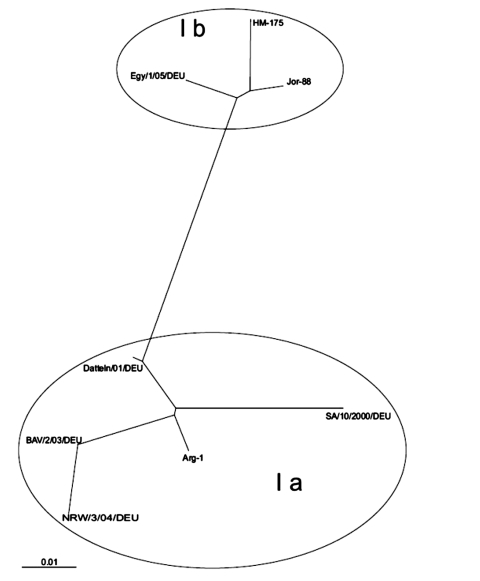
Phylogenetic analysis of the VP1–2A junction region. The sequence of a 160-bp-long PCR fragment (isolate Egy/1/05/DEU, GenBank accession no. AY741663) of the hepatitis A VP1 region was compared to published reference sequences of hepatis A virus. Genotype Ia: GenBank accession nos. AJ306374(Arg-1), AY656712 (BAV/2/03/DEU*), AY028976 (SA/10/2000/DEU), AY046073 (Datteln/01/DEU), and AY747173 (NRW/3/04/DEU*). Genotype Ib: L07728 (Jor 88), M14707 (HM-175). The scale represents nucleotide substitutions per position (* denotes previous German outbreak-causing strains).

Case-patients and controls did not differ significantly by age, sex, recreational activities, consumption of ice cream or salads, or other foods consumed or behavioral characteristics. Case-patients were significantly more likely to have drunk orange juice served at the breakfast buffet (82%) than were controls (64%) (odds ratio 2.6; 95% confidence interval 1.1–6.6). In multivariate analysis, no other exposures were retained. A dose-response relationship became apparent between number of days of orange juice consumption and HA (Figure 2). Case-patients consumed orange juice for a median of 11 days, and controls consumed it for a median of 5 days. In 22 (52%) of the 42 serum samples available for testing, HAV RNA was identified. All samples compared by sequencing were identical and belonged to genotype 1b (Figure 3).

The on-site investigations in Egypt did not identify hotel staff positive for HAV IgM. Minimal fluctuation among hotel staff renders it unlikely that an HAV-positive employee was missed. Investigations at the orange juice producing plant found significant hygiene problems. In addition, the finished product did not undergo heat treatment. This producer did not cater to other hotel chains in Hurghada.

## Conclusions

This large outbreak demonstrates risk and clinical impact of HA for nonimmune travelers to HA-endemic countries. In Germany, the outbreak accounted for 12% of all HA case-patients notified in the year 2004.

The results of the outbreak investigation strongly point to orange juice as the infection vehicle. In the case-control study, among a broad range of foodstuff, beverages, and recreational activities queried, the consumption of orange juice was the only exposure significantly associated with HA, with higher doses of juice significantly increasing HA risk. These findings are corroborated by the inspection of the hygienic conditions under which the juice was produced in Egypt. The juice was most likely contaminated during the manufacturing process, e.g., by an infected worker with imperfect hand hygiene or by contact of fruit or machinery with sewage-contaminated water.

Citrus fruit and citrus juices have only rarely been implicated as vehicles of HA outbreaks, with contamination typically described during preparation just before consumption (*4**,**5*). Salmonella outbreaks caused by orange juice contaminated at the production site have been identified (*6**–**8*). As HAV is quite resistant to acid (*9*), it likely survives for prolonged periods in orange juice. Less stable pathogens such as Escherichia coli have been shown to survive in orange juice for >15 days (*10*).

The fact that juice was consumed by 60% of healthy controls may be explained in part by fluctuating virus concentration within the juice, which resulted in varying degrees of infectiousness during the 4-week period. A contaminated lot may have been phased-in and out slowly by gradual mixture with other lots. Also, the study design did not allow the exclusion of controls who did not know they were immune.

The Hurghada outbreak-strain clearly differs from strains that have caused nontravel-associated outbreaks in Germany in recent years. Two large autochthonous outbreaks were caused by HAV type 1a strains (*11*). In the Netherlands, HAV strains in autochthonous cases mostly also belonged to HAV 1a, whereas 1b strains were found more often in children of Moroccan origin (*12*). Extended monitoring of HAV strains, for example, as performed in the United States (*13*), could find hidden clusters and demonstrate links between imported and autochthonous cases. Similar monitoring should be introduced in Germany.

Vaccination against HA is recommended for all nonimmune travelers to HA-endemic areas (*14*). This outbreak showed that a high proportion of German tourists in Egypt were either not adequately informed about HA risk and the benefits of vaccination or were informed yet still decided against vaccination. The outbreak emphasizes the importance of adequate pretravel advice, preferably from an institution specialized in travel medicine. Travel agencies should incorporate adequate immunization advice into their catalogs.
